# Does Counterclockwise Rotation Affect Fixation Stability? An In Vitro Biomechanical Study of Large Mandibular Advancements

**DOI:** 10.3390/bioengineering13070745

**Published:** 2026-06-26

**Authors:** Beethoven Estevão Costa, Maísa Pereira-Silva, Bianca Tiemi Uehara Lima, Gustavo Batista Grolli Klein, Celso Fernando Palmieri Junior, Daniel Oreadi, Luana Ferreira Oliveira, Paulo Matheus Honda Tavares, Paulo Domingos Ribeiro Junior, Osvaldo Magro Filho

**Affiliations:** 1 Department of Diagnosis and Surgery, School of Dentistry, São Paulo State University (UNESP), Araçatuba 16010-380, SP, Brazil; beethoven.e.costa@unesp.br (B.E.C.); bianca.tiemi@unesp.br (B.T.U.L.); celso.palmieri@lsuhs.edu (C.F.P.J.); paulo.matheus@unesp.br (P.M.H.T.); 2 Department of Oral and Maxillofacial Surgery, São Leopoldo Mandic, Campinas 13045-755, SP, Brazil; gutoklein@yahoo.com.br; 3 Department of Oral and Maxillofacial Surgery, Louisiana State University Health Shreveport (LSUHS), Shreveport, LA 71103, USA; 4 Department of Oral and Maxillofacial Surgery, Tufts University School of Dental Medicine, Boston, MA 02111, USA; daniel.oreadi@tufts.edu; 5 Department of Oral and Maxillofacial Surgery, Santa Casa de Jahu, Jaú 17201-340, SP, Brazil; paulodominos@iocp.com.br

**Keywords:** osteotomy, Internal fracture fixation, mandible, orthognathic surgery

## Abstract

The selection of an appropriate fixation system is critical for maintaining postoperative stability after sagittal split ramus osteotomy (SSRO), especially in cases involving large mandibular advancements and counterclockwise rotation, where mechanical stresses may compromise treatment outcomes. This in vitro study evaluated the biomechanical stability of five fixation systems following sagittal split ramus osteotomy (SSRO) under two mandibular advancement conditions. Fifty polyurethane hemimandibles were allocated into two experimental groups: Group 1, submitted to 10-mm linear advancement, and Group 2, submitted to 10-mm advancement associated with 20° counterclockwise rotation. Each group was further divided into five subgroups according to the fixation design employed: (A) conventional straight plate, (B) angled plate, (C) sagittal plate, (D) 10-hole miniplate, and (E) two 4-hole miniplates. Biomechanical performance was assessed by compression testing using a universal testing machine. Complementary analyses were performed using strain gauges and digital image correlation. Group 1 in fixation B, C and D demonstrated higher biomechanical resistance than Group 2, however without significance difference. Among the evaluated configurations, subgroup E exhibited the highest resistance values in both experimental conditions. Statistically significant differences were observed among fixation systems within each group (*p* < 0.05). The fixation systems using two miniplates demonstrated superior biomechanical stability, particularly in mandibular advancements associated with counterclockwise rotational movements. These findings suggest that this fixation strategy may contribute to enhanced postoperative stability in orthognathic surgery involving large mandibular advancements and complex rotational movements.

## 1. Introduction

Sagittal split ramus osteotomy (SSRO) is one of the most commonly used surgical techniques for correcting dentofacial skeletal deformities [1,2,3]. The postoperative stability of this procedure depends on several factors, among which the type of fixation employed is crucial. An inadequate choice of fixation system may lead to complications such as relapse, lack of stability, and condylar displacement, thus compromising surgical success and the functional recovery of the patient [4,5,6].

Fixation systems based on bicortical screws and miniplates have been widely investigated in both laboratory and clinical studies, demonstrating superior performance in stabilizing bone segments [7,8,9]. However, the ongoing development of materials and surgical techniques necessitates a deeper biomechanical evaluation of these approaches. Factors such as the magnitude of mandibular advancement and the presence of asymmetries may influence the effectiveness of fixation systems, making it essential to conduct comparative studies analyzing different fixation protocols [10,11].

Additionally, new approaches using resorbable materials have been studied as viable alternatives to titanium systems, focusing on reducing long-term complications such as infections and the need for hardware removal [12,13]. Recent studies highlight biomechanical differences between these materials, particularly in terms of compressive strength and structural rigidity [14].

Preclinical biomechanical studies play a crucial role in validating fixation devices by simulating the mechanical conditions to which the mandible is subjected postoperatively. Among the methods used for this evaluation, compression testing, strain gauge analysis, and 3D computational vision stand out [15,16]. These experimental models provide a better understanding of the stresses generated in fixation systems and their impact on bone stability. Thus, this study aimed to evaluate the biomechanical performance of five different fixation systems applied to two distinct mandibular advancement movements in SSRO.

## 2. Materials and Methods

A total of 50 polyurethane hemimandibles (model CHF 43.75; Synbone, Zizers, Switzerland), with bone-like consistency and containing cortical and medullary layers, were used. Sample allocation was established according to the methodology adopted in a previous biomechanical investigation evaluating fixation techniques following sagittal split ramus osteotomy, which used the same number of specimens per subgroup and demonstrated adequate discrimination of mechanical differences between fixation systems [17]. The hemimandibles were randomly divided into two groups (n = 25), according to the type of mandibular movement performed. Each group was further subdivided into five subgroups (n = 5), based on the fixation system used.

**Figure 1:**
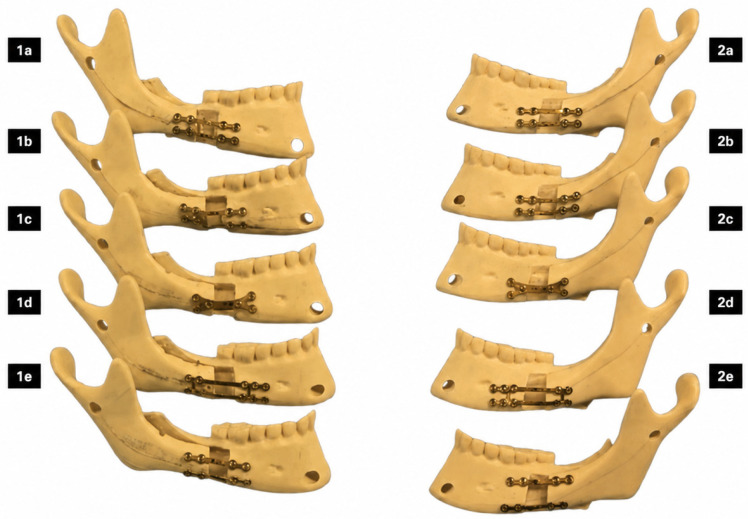
Group 1 specimens (**1a**–**1e**) with subgroups, demonstrating the fixation in linear advancement of 10 mm. Group 2 specimens (**2a**–**2e**) with subgroups, in which the plates were positioned after a 10-mm mandibular advancement was combined with a 20° counterclockwise rotation.

Sagittal mandibular osteotomies were performed according to the modified technique described by Epker [18]. In Group 1, a linear advancement of 10 mm was performed, without rotation, simulating classic cases of mandibular advancement for sagittal discrepancy correction. In Group 2, a 10-mm mandibular advancement was combined with a 20° counterclockwise rotation, simulating clinical situations that require adjustments to mandibular inclination, with an impact on the condylar position and postoperative stability (Figure 1) and (Table 1). This movement resulted in a displacement of 9 mm at the superior border and 12 mm at the mandibular base.

**Figure 2:**
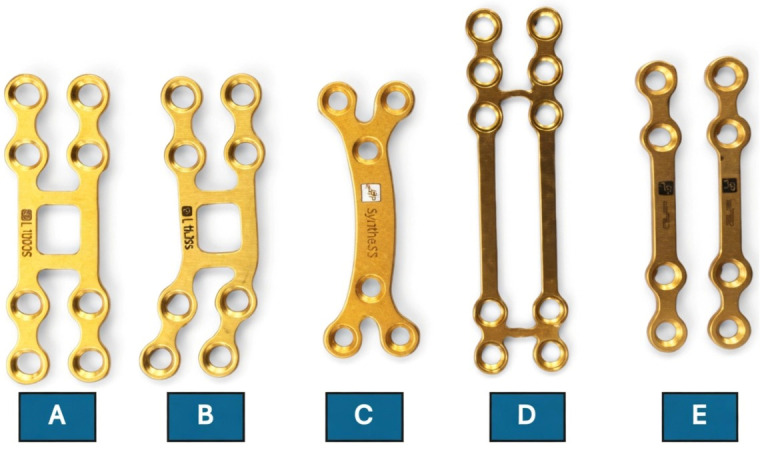
Examples of the fixation plates used in each subgroup: (**A**) conventional straight plate; (**B**) angled plate; (**C**) sagittal plate; (**D**) 10-hole miniplate; (**E**) two 4-hole miniplates.

Fixation was performed using 2.0 mm plates and monocortical screws (2.0 × 6 mm) (NeoOrtho, Curitiba, PR, Brazil), with the following subgroups: Subgroup A (1A/2A): A conventional straight plate with a simple double interval, fixed with eight monocortical screws; Subgroup B (1B/2B): An angulated plate with a double interval, fixed with eight monocortical screws; Subgroup C (1C/2C): A conventional sagittal plate, fixed with six monocortical screws; Subgroup D (1D/2D): A 10-hole miniplate with a double interval, with six screws in the distal segment and four in the proximal segment; Subgroup E (1E/2E): Two 4-hole miniplates with a long interval, with four screws in each segment (Figure 2). Plate positioning and osteotomy preparation were standardized using prefabricated surgical templates and previously established reference points to ensure reproducibility and sample uniformity.

### 2.1. Biomechanical Testing Protocol

**Figure 3:**
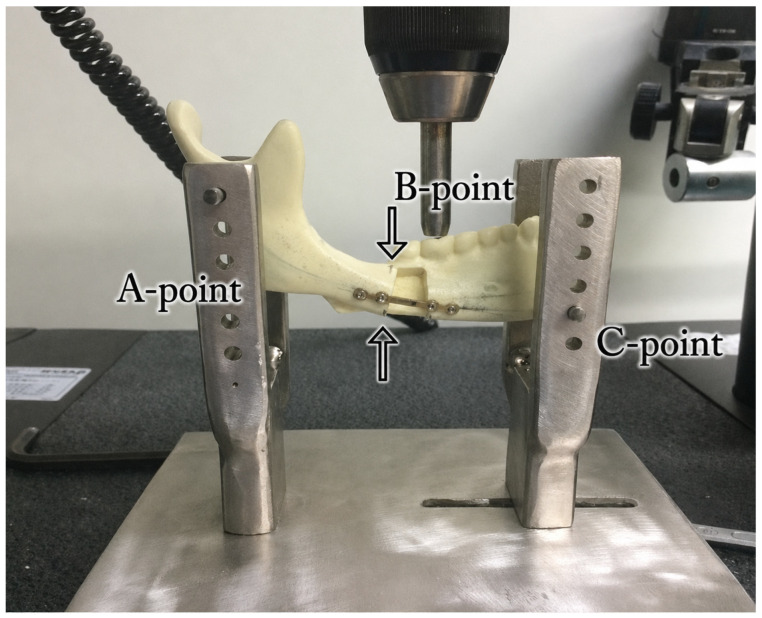
Polyurethane hemimandible mounted in the testing apparatus. Compressive forces were applied at the B-point using an Instron universal testing machine, while the mandible was stabilized at the A-point, simulating posterior condylar resistance, and at the C-point, representing the resultant anterior masticatory force.

Biomechanical analysis was performed using compression tests in a universal testing machine (model 4202; Instron, Norwood, MA, USA), following the method described by Ribeiro-Junior et al. [9] (Figure 3). The samples were mounted on a base developed specifically for the experiment, simulating the posterior condylar resistance and the resultant anterior masticatory forces. The compressive load was applied to the first molar region until a 3-mm displacement occurred between the mandibular segments, in both vertical and horizontal directions. The final load value was recorded in kN.

Displacements were measured at two distinct regions: the lower border of the mandible (vertical displacement) and the alveolar ridge in the second molar region (horizontal displacement), allowing for a three-dimensional biomechanical analysis. To ensure precise measurement of the 3-mm displacement, perforations were made on the segments, and the distances were verified with a surgical caliper before and during the test.

### 2.2. Statistical Analysis

Data normality was assessed using the Shapiro–Wilk test (α = 0.05). Intergroup comparisons were performed using the independent-samples *t*-test or Mann–Whitney U test, as appropriate. Comparisons among fixation subgroups were conducted using the Kruskal–Wallis test followed by Dunn’s post hoc test with Bonferroni correction. A significance level of 5% (*p* < 0.05) was adopted.

## 3. Results

### 3.1. Compressive Load to Failure

Biomechanical resistance values varied according to the fixation design in both experimental groups. In Group 1, Subgroup E demonstrated the highest mean resistance (0.2132 ± 0.02605 kN), followed by Subgroups D (0.1368 ± 0.03232 kN), B (0.1332 ± 0.02315 kN), C (0.1260 ± 0.01675 kN), and A (0.1204 ± 0.01899 kN).

**Figure 4:**
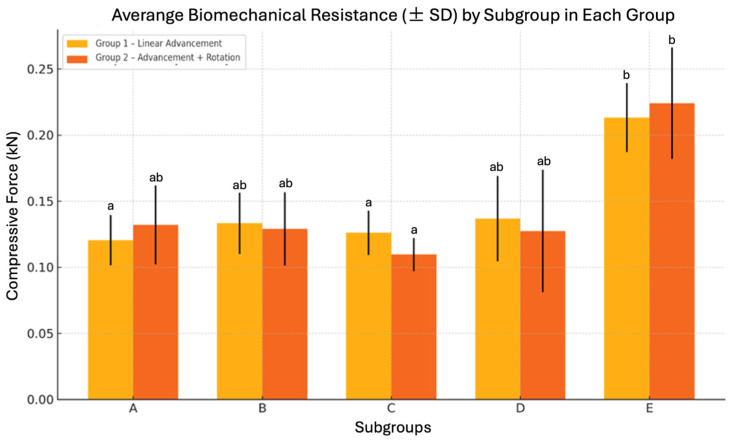
Mean biomechanical resistance (±SD) for each fixation subgroup in Groups 1 and 2, expressed as maximum compressive force (kN). Different lowercase letters indicate statistically significant differences among fixation subgroups (*p* < 0.05), within the same experimental group.

A similar pattern was observed in Group 2, in which Subgroup E also showed the highest mean resistance (0.2240 ± 0.04216 kN), followed by Subgroups A (0.1320 ± 0.02978 kN), B (0.1290 ± 0.02783 kN), D (0.1274 ± 0.04624 kN), and C (0.1096 ± 0.01254 kN) (Figure 4).

Statistical analysis revealed significant differences among fixation subgroups within both experimental groups (Group 1: *p* = 0.006; Group 2: *p* = 0.007). However, no statistically significant differences were observed between Groups 1 and 2 within the same fixation subgroup (*p* > 0.05).

The Kruskal–Wallis test revealed significant differences among fixation subgroups in both Group 1 (*p* = 0.006) and Group 2 (*p* = 0.007). As illustrated in Figure 4 Subgroup E demonstrated the highest biomechanical resistance values in both experimental conditions, whereas the remaining subgroups showed relatively similar mean values.

### 3.2. Displacement Measurements

**Figure 5:**
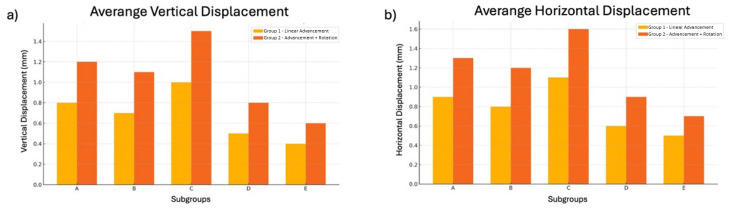
(**a**) Mean vertical displacement (mm) by subgroup. (**b**) Mean horizontal displacement (mm) by subgroup.

Group 2 tended to exhibit greater vertical and horizontal displacement values compared with Group 1. Vertical displacement was highest in Subgroup C (1.5 mm), followed by Subgroups A (1.2 mm), B (1.1 mm), D (0.8 mm), and E (0.6 mm) (Figure 5a).

Horizontal displacement followed a similar pattern, with Subgroup C presenting the highest displacement values (1.6 mm), followed by Subgroups A (1.3 mm), B (1.2 mm), E (1.0 mm), and D (0.9 mm) (Figure 5b).

These findings suggest that fixation systems using two plates or longer plates with multiple screw distribution (Subgroup E) provide greater biomechanical stability in both linear and rotational mandibular advancements. Statistical analysis revealed significant differences among fixation subgroups within each experimental group (Group 1: *p* = 0.006; Group 2: *p* = 0.007). However, no statistically significant differences were observed between Group 1 and Group 2 within the same fixation subgroup (*p* > 0.05 for all comparisons), suggesting that fixation design may exert greater influence on biomechanical stability than the mandibular movement pattern itself.

## 4. Discussion

Achieving stable skeletal outcomes following sagittal split ramus osteotomy (SSRO) remains a major challenge in orthognathic surgery, particularly in cases involving large mandibular advancements and counterclockwise rotation. These movements increase the magnitude and complexity of mechanical forces acting across the osteotomy site, potentially compromising postoperative stability and increasing the risk of relapse. In this context, the fixation system plays a critical role by providing mechanical support during bone healing and by controlling displacement generated by functional loading. Differences in plate design, screw distribution, and overall osteosynthesis configuration may alter stress distribution and construct rigidity, thereby influencing the ability of the fixation system to maintain the planned skeletal position. Therefore, understanding the biomechanical behavior of different fixation strategies is essential for optimizing treatment outcomes and minimizing postoperative complications.

The present study demonstrated that the biomechanical behavior of fixation systems in simulated mandibular advancements is strongly influenced by the configuration of the osteosynthesis. Among all fixation models evaluated, the dual-plate configuration (Subgroup E) consistently exhibited the highest resistance to compressive loading and the lowest displacement values in both linear and rotational advancement models. In contrast, the conventional sagittal plate configuration (Subgroup C) showed the poorest mechanical performance, particularly under rotational loading conditions.

From a biomechanical perspective, fixation systems with broader screw distribution and multiple stabilization points appear to provide more effective dissipation of multidirectional forces generated during mandibular advancement and counterclockwise rotation. This effect became more evident in Group 2, where rotational movement increased instability and displacement in most fixation models. Nevertheless, Subgroup E maintained superior resistance and reduced displacement, suggesting enhanced structural rigidity and improved force distribution.

These findings are consistent with previous biomechanical investigations demonstrating that dual-plate, bicortical, and hybrid fixation systems provide greater resistance and improved stress distribution compared with conventional monocortical miniplate techniques [19,20,21,22,23,24,25,26,27]. Previous in vitro and finite element studies have shown that increasing the number and spatial distribution of fixation points reduces stress concentration on both fixation hardware and surrounding cortical bone, thereby improving overall mechanical stability [20,22,23,24,25]. Similarly, studies evaluating hybrid fixation techniques have reported superior resistance under rotational and high-load conditions, reinforcing the importance of fixation geometry and screw distribution in maintaining postoperative stability [24,27]. Recent finite element and experimental studies further confirmed that fixation systems with broader spatial screw distribution and increased rigidity significantly reduce displacement and stress concentration during mandibular advancement procedures, particularly under high-load and rotational conditions [28,17]. In addition, computational analyses demonstrated that conventional single miniplate systems tend to concentrate stress around the osteotomy region and fixation screws, whereas dual-plate and hybrid configurations promote more homogeneous load distribution and improved biomechanical stability [17,28].

Clinically, these findings suggest that more rigid fixation systems may be advantageous in cases involving large mandibular advancements and rotational movements, where postoperative instability and relapse remain significant concerns. Although the present study was limited to an in vitro mechanical model, the results support the concept that optimized load distribution and enhanced structural rigidity are critical factors for improving fixation performance in sagittal split ramus osteotomy procedures.

Although Group 2 exhibited greater displacement values overall, no statistically significant differences were observed between Groups 1 and 2 within the same fixation subgroup. These findings suggest that fixation design may exert greater influence on biomechanical stability than the mandibular movement pattern itself, even under rotational loading conditions. Collectively, the present findings reinforce the importance of fixation geometry and screw distribution in the biomechanical stability of sagittal split ramus osteotomy, particularly during high-demand movements involving rotational components.

Beyond static biomechanical performance, future investigations may benefit from incorporating emerging technologies capable of objectively assessing mandibular function and movement patterns after orthognathic surgery. Recent study demonstrated the high accuracy of a novel photometric jaw-tracking system for recording mandibular movements in the frontal plane, highlighting its potential application for quantitative functional assessment [29]. Although the present study focused on the mechanical behavior of different fixation systems under controlled laboratory conditions, integrating biomechanical analyses with advanced jaw-tracking technologies may provide a more comprehensive understanding of postoperative stability. Such approaches could help correlate fixation rigidity and displacement resistance with dynamic mandibular function, offering valuable insights into the clinical performance of osteosynthesis systems following sagittal split ramus osteotomy.

The primary limitation of this study is the use of polyurethane hemimandibles, which do not fully reproduce the biological and viscoelastic characteristics of human bone tissue. Furthermore, the experimental model did not account for the influence of surrounding soft tissues, muscular forces, bone healing, or postoperative remodeling, all of which may affect the clinical behavior of fixation systems. Another limitation is that only static compressive loading was evaluated, whereas masticatory function subjects the mandible to complex multidirectional and cyclic forces over time.

Another important consideration is that the loading conditions applied in the present study do not fully reproduce the magnitude and variability of forces observed clinically. While normal masticatory activity generates functional loads that are generally compatible with the stability of contemporary fixation systems, parafunctional habits such as bruxism may subject the mandible to substantially greater and repetitive forces, potentially increasing the risk of fixation overload and postoperative instability. Therefore, fixation systems exhibiting greater rigidity and more favorable load distribution may be particularly beneficial under high-demand functional conditions. Furthermore, advances in digital manufacturing technologies have expanded the development of experimental models and biomaterials for dental applications. A recent systematic review demonstrated the growing relevance of additively manufactured and CAD-CAM polymeric materials, highlighting their promising mechanical properties for clinical and research applications [30]. Nevertheless, standardized polyurethane models remain widely accepted in maxillofacial biomechanical investigations because of their reproducibility, homogeneous mechanical behavior, and ability to provide reliable comparisons between fixation systems.

In addition, the use of isolated hemimandibles and a limited sample size per subgroup may restrict the direct extrapolation of the findings to clinical conditions. Future investigations should incorporate cyclic fatigue testing and more physiologic loading conditions to better simulate postoperative functional demands and improve the clinical applicability of the findings.

## 5. Conclusions

Within the limitations of this in vitro study, fixation geometry and screw distribution appear to exert greater influence on biomechanical stability than the mandibular movement pattern itself. Dual-plate configurations demonstrated superior biomechanical performance and reduced displacement values under both linear and counterclockwise rotational advancement conditions. These findings reinforce the importance of optimized fixation design in sagittal split ramus osteotomy, particularly in high-demand mandibular movements involving rotational components.

## Figures and Tables

**Table 1:** Distribution of experimental groups according to mandibular movement pattern, fixation system, and sample size. Group 1 consisted of specimens submitted to 10-mm mandibular advancement, whereas Group 2 consisted of specimens submitted to 10-mm mandibular advancement combined with 20° counterclockwise (CCW) rotation. Each fixation subgroup included five hemimandibles.

Group	Mandibular Movement	Fixation System	n
G1A	10-mm advancement	Conventional straight plate	5
G1B	10-mm advancement	Angled plate	5
G1C	10-mm advancement	Sagittal plate	5
G1D	10-mm advancement	10-hole miniplate	5
G1E	10-mm advancement	Two 4-hole miniplates	5
G2A	10-mm advancement + 20° CCW rotation	Conventional straight plate	5
G2B	10-mm advancement + 20° CCW rotation	Angled plate	5
G2C	10-mm advancement + 20° CCW rotation	Sagittal plate	5
G2D	10-mm advancement + 20° CCW rotation	10-hole miniplate	5
G2E	10-mm advancement + 20° CCW rotation	Two 4-hole miniplates	5

## Data Availability

Data supporting the findings of this study are available from the corresponding author upon reasonable request.

## Abbreviations

The following abbreviations are used in this manuscript:

SSRO	Sagittal split ramus osteotomy
kN	Kilonewton
